# Comprehensive analysis of ceRNA Networks in UCEC: Prognostic and therapeutic implications

**DOI:** 10.1371/journal.pone.0314314

**Published:** 2025-01-30

**Authors:** Li Fan, Mengqiu Lan, Xiaohua Wei, Lili Wei, Liuhong Yang, Liuying Nong, Jiajia Wei, Jingjing Li, Wenjie Huang

**Affiliations:** 1 Department of Reproductive Medicine, Guangzhou Women and Children’s Medical center Liuzhou Hospital, Liuzhou, Guangxi, China; 2 Reproductive Medicine Center, Liuzhou Maternity and Child Health Care Hospital, Liuzhou, China; 3 Guangxi Maternal and Obstetric Disease Research Center, Liuzhou, China; 4 Liuzhou Institute of Reproduction and Genetics, Liuzhou Maternity and Child Health Care Hospital, Liuzhou, China; 5 Liuzhou Key Laboratory of Gynecologic Tumor, Zhengzhou, China; 6 Liuzhou Municipal Liutie Central Hospital, Liuzhou, Guangxi, China; BMSCE: BMS College of Engineering, INDIA

## Abstract

Endometrial cancer (UCEC) is the most prevalent gynecological malignancy in high-income countries, and its incidence is rising globally. Although early-stage UCEC can be treated with surgery, advanced cases have a poor prognosis, highlighting the need for effective molecular biomarkers to improve diagnosis and prognosis. In this study, we analyzed mRNA and miRNA sequencing data from UCEC tissues and adjacent non-cancerous tissues from the TCGA database. Differential expression analysis was conducted using the DESeq2 package, identifying differentially expressed lncRNAs, miRNAs, and mRNAs (DElncRNAs, DEmiRNAs, and DEmRNAs). Key molecules were screened using LASSO regression, and a ceRNA network was constructed by predicting lncRNA-miRNA and miRNA-mRNA interaction, which were visualized with Cytoscape. Functional enrichment analysis elucidated the roles and mechanisms of the network. The prognostic potential of the identified RNAs was assessed through survival and Cox regression analyses, while methylation and immune infiltration analyses explored regulatory mechanisms and immune interactions. We identified a prognostic lncRNA-miRNA-mRNA ceRNA network in UCEC, centered on the CDKN2B-AS1-hsa-miR-497-5p-IGF2BP3 axis. Survival analyses confirmed the prognostic significance of this network, with univariate Cox regression demonstrating a strong association between its aberrant expression and overall prognosis in UCEC. However, multivariate Cox regression suggested that other clinical factors may modulate this relationship. Methylation analysis revealed low methylation levels of IGF2BP3, possibly contributing to its overexpression. Furthermore, immune infiltration studies highlighted significant correlations between CDKN2B-AS1, IGF2BP3, and multiple immune cell types, suggesting that this axis regulates the tumor immune microenvironment. These findings suggest that the CDKN2B-AS1-hsa-miR-497-5p-IGF2BP3 axis is a key regulatory element in UCEC and a potential therapeutic target.

## Introduction

Endometrial cancer (UCEC) is the most common gynecological cancer in high-income countries, with its incidence steadily rising worldwide [1]. In 2020, there were approximately 417,000 new cases diagnosed globally, with a woman’s lifetime risk of developing endometrial cancer estimated at around 3% [2]. Although early-diagnosed UCEC can typically be cured through surgery, the prognosis for advanced cases remains poor. The median age at diagnosis is 61 years. Over the past 30 years, the incidence of UCEC has increased by 132%, largely due to obesity and an aging population [3]. However, women in low- and middle-income countries face a higher risk of mortality from UCEC due to limited access to timely medical care [1]. Given the high incidence and mortality rates of UCEC, there is an urgent need for effective molecular biomarkers to improve its diagnosis and prognosis.

In recent years, research on tumorigenesis and cancer progression has unveiled numerous novel molecular mechanisms, particularly the interactions between cellular non-coding RNAs (such as lncRNAs and miRNAs) and coding transcripts, which play significant regulatory roles in both health disease. Studies have shown that the interactions and dysregulation of lncRNAs, miRNAs, and mRNAs play a crucial role in the pathophysiology of UCEC [4]. Many lncRNAs can act as molecular sponges [5]. sequestering various tumor-suppressive miRNAs, thereby inhibiting their function and leading to the dysregulation of their target mRNA transcripts, affecting UCEC regulation.

LncRNAs and miRNAs are two major classes of non-coding RNAs that play important roles in various cancer processes, such as cell proliferation, metastasis, drug resistance, and cancer stem cell initiation [6]. By regulating gene expression, lncRNAs and miRNAs can not only reveal the molecular mechanisms of UCEC but also provide new diagnostic biomarkers and therapeutic intervention points, aiding in the improvement of UCEC treatment outcomes. For example, the interaction between MALAT1 and miR-200c is considered a key factor in regulating the epithelial-mesenchymal transition (EMT) process in UCEC [7]. MALAT1 promotes EMT and tumor growth by sequestering miR-200c. Similarly, NEAT1 interacts with miR-361 and miR-144-3p to regulate the expression of STAT3 and EZH2, promoting the proliferation and invasion of UCEC cells [8,9]. Additionally, lncRNAs such as SNHG5 and TTN-AS1 also influence the progression of UCEC by sequestering specific miRNAs and regulating the expression of their target mRNAs [10,11]. These findings indicate that the complex regulatory network between lncRNAs, miRNAs, and mRNAs plays an essential role in the pathogenesis of UCEC. By further investigating these molecular mechanisms, we can gain a better understanding of the initiation and progression of UCEC, providing new avenues and strategies for early diagnosis and personalized treatment.

In our study, we analyzed mRNA sequencing data from 554 UCEC cases and 35 adjacent non-cancerous tissues, as well as miRNA sequencing data from 546 UCEC cases and 33 adjacent non-cancerous tissues. Using the DESeq2 package for differential expression analysis, we identified DElncRNAs, DEmiRNAs, and DEmRNAs. Key molecules were screened through LASSO regression, and their interactions were revealed by constructing a ceRNA network. Functional enrichment analysis was performed to elucidate the functional roles and potential mechanisms of this network in UCEC. Through detailed analyses of key RNAs, survival rates, subcellular localization, and related studies, we identified a critical CDKN2B-AS1-hsa-miR-497-5p-IGF2BP3 ceRNA network. Further investigation using Cox regression analysis revealed the diagnostic and prognostic potential of the CDKN2B-AS1-hsa-miR-497-5p-IGF2BP3 axis in UCEC. Additionally, we explored the role of IGF2BP3 in UCEC through methylation analysis, immune infiltration studies, and GO and KEGG pathway analyses to understand the potential functions of IGF2BP3 and its associated binding proteins in UCEC.

The aim of this study is to construct and validate a ceRNA network in UCEC, identify key regulatory RNAs as potential diagnostic and prognostic biomarkers, and explore their molecular mechanisms, including roles in immune regulation and epigenetic modifications.

## Materials and methods

### Data preparation and preprocessing

We obtained mRNA sequencing (mRNA-seq) data for 554 UCEC cases and 35 adjacent non-cancerous samples. and miRNA sequencing (miRNA-seq) data for 546 UCEC cases and 33 adjacent non-cancerous samples from the TCGA database (https://portal.gdc.cancer.gov/). Survival information for the TCGA-UCEC dataset was sourced from UCSC Xena (http://xena.ucsc.edu/). The publicly available data were divided into cancerous and adjacent non-cancerous groups. We performed differential expression analysis on the raw count matrices of the selected public data using the DESeq2 package, following standard procedures. Additionally, we applied the variance stabilizing transformation (VST) method provided by the DESeq2 package to normalize the raw count matrices. Protein expression analysis was conducted through the UALCAN portal (http://ualcan.path.uab.edu/analysis-prot.html).

### Identification of differentially expressed lncRNAs, mRNAs, and miRNAs

The identification of differentially expressed lncRNAs (DElncRNAs) was performed using a threshold of |logFC| > 1 and p.adj < 0.05. For differentially expressed mRNAs (DEmRNAs), the threshold ws set at |logFC| > 2 and p.adj < 0.05. Differentially expressed miRNA (DEmiRNA) were identified using a threshold of |logFC| > 1 and p.adj < 0.05. Volcano plot visualizations of differentially expressed genes (DEGs) were generated using the R package "EnhancedVolcano". Heatmap clustering of DEGs was performed using the R package "pheatmap".

### Survival regression analysis

We performed bilk survival regression analysis using the "survival" package to identify RNAs associated with survival. RNAseq data were downloaded and organized form the TCGA-UCEC project using the STAR pipeline in the TCGA database. We extracted data in TPM (Transcripts Per Million) format along with clinical data. Supplementary data, including prognostic information, were sourced from a previous study by Liu et al. [12]. The data filtering strategy involved removing normal samples and samples lacking clinical information. The data were processed using the transformation log2(value+1). Bulk fitting of survival regression was performed using the survival package. Statistical analysis was conducted using Cox regression, with p-values adjusted using the Bonferroni correction method. The grouping strategy was defined as 0–50 vs. 50–100.

We performed univariate and multivariate Cox regression analyses to assess the correlation of candidate genes in the ceRNA network with Overall Survival (OS), Disease-Specific Survival (DSS), and Progression-Free Interval (PFI), aiming to identify UCEC prognostic biomarkers and independent prognostic factors. Statistical significance was set at p < 0.05. Additionally, we conducted stratified analyses to determine whether the prognostic value of these biomarkers remained stable across different subgroups.

### ROC analysis

Based on the expression levels of candidate genes, we conducted Receiver Operating Characteristic (ROC) analysis using the "pROC" package to evaluate the predictive ability of biomarkers and calculate the Area Under the Curve (AUC). The results were visualized using the "ggplot2" package. When AUC >0.5, the closer the AUC is to 1, the better the variable’s diagnostic effectiveness in predicting the outcome. An AUC between 0.5–0.7 indicates low accuracy, an AUC between 0.7 and 0.9 indicates moderate accuracy, and an AUC above 0.9 indicates high accuracy.

### Machine learning

We will further screen the intersecting lncRNAs, which are both differentially expressed in UCEC and associated with UCEC survival, and the intersecting miRNAs, which are both differentially expressed and associated with survival, using Least Absolute Shrinkage and Selection Operator (LASSO) regression with the ’glmnet’ R package. LASSO regression helps in variable selection and regularization, improving the predictive performance and interpretability of the model.

### Construction of the ceRNA

Based on the hypothesis that lncRNAs act as natural sponges in the cytoplasm, indirectly regulating mRNA expression through competitive binding with miRNAs, the construction steps of the ceRNA network are as follows: 1) Utilize miRNet (https://www.mirnet.ca) to predict potential miRNAs targeted by prognostic lncRNAs and to identify lncRNA-miRNA interactions. 2) Use TargetScan (http://www.targetscan.org) and miRDB (http://www.mirdb.org) to predict target mRNAs by intersecting the predicted miRNAs with prognostic miRNAs. 3) Use the R package "VennDiagram" to obtain the intersecting mRNAs from DEmRNAs, OS-related mRNAs, and predicted target mRNAs for subsequent analysis. 4) Integrate lncRNA-miRNA and miRNA-mRNA pairs to construct the lncRNA-miRNA-mRNA triple regulatory network. The generated network is visualized using Cytoscape software.

The subcellular localization of lncRNAs related to experimental evidence is determined by retrieving data from RNALocate [13]. We performed functional similarity analysis using the GOSemSim package. The positions of the target genes on the chromosomes were mapped using the "circlize" package.

### Functional enrichment analysis and visualization

We performed functional enrichment analysis on the mRNAs in the lncRNA-miRNA-mRNA triple regulatory network using R packages "Goplot" and "clusterProfiler". The significance threshold for GO terms and KEGG pathways [14] was set at p.adj < 0.05. Additionally, we calculated the pairwise similarity of enriched terms using the Jaccard similarity index (JC) and performed clustering analysis on the results using hclust. The clustering results were visualized using the ggplot2 package.

Furthermore, we obtained a set of 35 experimentally validated IGF2BP3 binding proteins from the STRING database (https://string-db.org). We analyzed the correlation of these genes in UCEC and visualized the correlation results using the "circlize" package. We then performed Go enrichment analysis (Molecular Function; MF, Cellular Component; CC, Biological Process; BP) and visualized the results with the "ggplot2" package.

Based on the UniProt website, we obtained domain annotations for these proteins using their IDs and conducted domain enrichment analysis the hypergeometric distribution.

### Analysis of DNA methylation and expression

DNA methyltransferases (DNMT1, DNMT3A, and DNMT3B) are key player epigenetic regulation, with roles ranging from maintaining existing DNA methylation patterns to establishing new ones. Their impact on gene expression and epigenetic memory makes them important subjects of subjects of study in molecular biology and genetics. We investigated the expression levels of these three DNA methyltransferases in IGF2BP3 high and low groups using data from the TCGA database.

Additionally, we compared the methylation levels of IGF2BP3 between UCEC and adjacent normal tissues using DiseaseMeth version 3.0 (http://bio-bigdata.hrbmu.edu.cn/diseasemeth/). We used MEXPRESS (https://mexpress.ugent.be) to display the methylation levels of different CpG sites within the IGF2BP3 gene and to study the relationship between DNA methylation and gene expression levels.

MethSurv (https://biit.cs.ut.ee/methsurv/) was employed to analyze the methylation levels of IGF2BP3 CpG sites in UCEC and to examine the correlation between these CpG sites and the survival of endometrial cancer patients. Finally, we used SMART (http://www.bioinfo-zs.com/smartapp/) to investigate the methylation values of different CpG sites of IGF2BP3 in UCEC and normal tissues.

### Correlation analysis of gene expression and UCEC risk

We identified key SNPs that affect IGF2BP3 expression using the GTEx Portal (https://gtexportal.org/home/singleCellOverviewPage). The UCEC data was sourced from genome-wide association study (GWAS), which included a European cohort of 2,188 patients and 237,839 control participants [15]. Using the " TwoSampleMR" package, we explored the correlation between SNPs’ normalized expression scores and the risk endometrial cancer.

### Analysis of immune infiltration and potential therapeutic agents in UCEC

We utilized the core algorithm of CIBERSORT via the CIBERSORTx website (https://cibersortx.stanford.edu/) to calculate immune infiltration using markers of 22 immune cell types [16]. The ssGSEA algorithm provided by the "GSVA" R package was employed to compute the immune infiltration status of UCEC using markers of 24 immune cell types identified by Bindea et al. [17]. We analyzed the correlation between CDKN2B-AS1, IGF2BP3, and these immune cells, and visualized the results using the "ggplot2" package.

Additionally, based on the median expression levels of CDKN2B-AS1 and IGF2BP3 in UCEC samples, we classified the samples into high and low expression groups. This classification allowed us to analyze the differences in immune infiltration between the two groups. Moreover, we obtained drugs interacting with the candidate diagnostic genes from the Comparative Toxicogenomics Database (CTD, https://ctdbase.org) to predict potential therapeutic agents for UCEC. The 3D structures of these drugs were displayed using the ChemSpider website (https://www.chemspider.com/).

#### Ethics statement

This study used publicly available data from TCGA database, which is already anonymized and de-identified. As such, no additional ethical approval or informed consent was required for the use of this data.

### Statistical analysis

All data analyses and visualizations in this study were conducted using R software (version 4.2.1; https://www.r-project.org/) along with appropriate R packages. Differential expression analysis was performed using DESeq2 with Benjamini-Hochberg FDR correction to adjust for multiple comparisons. Prognostic significance was assessed using univariate and multivariate Cox regression, adjusting for clinical factors such as age, stage, and treatment outcome. ROC analysis was used to evaluate the predictive performance of biomarkers, with AUC values interpreted based on established criteria. A significance threshold of p < 0.05 was applied.

## Results

### Identification of differentially expressed genes (DEGs) between UCEC and adjacent normal samples

**Fig 1:**
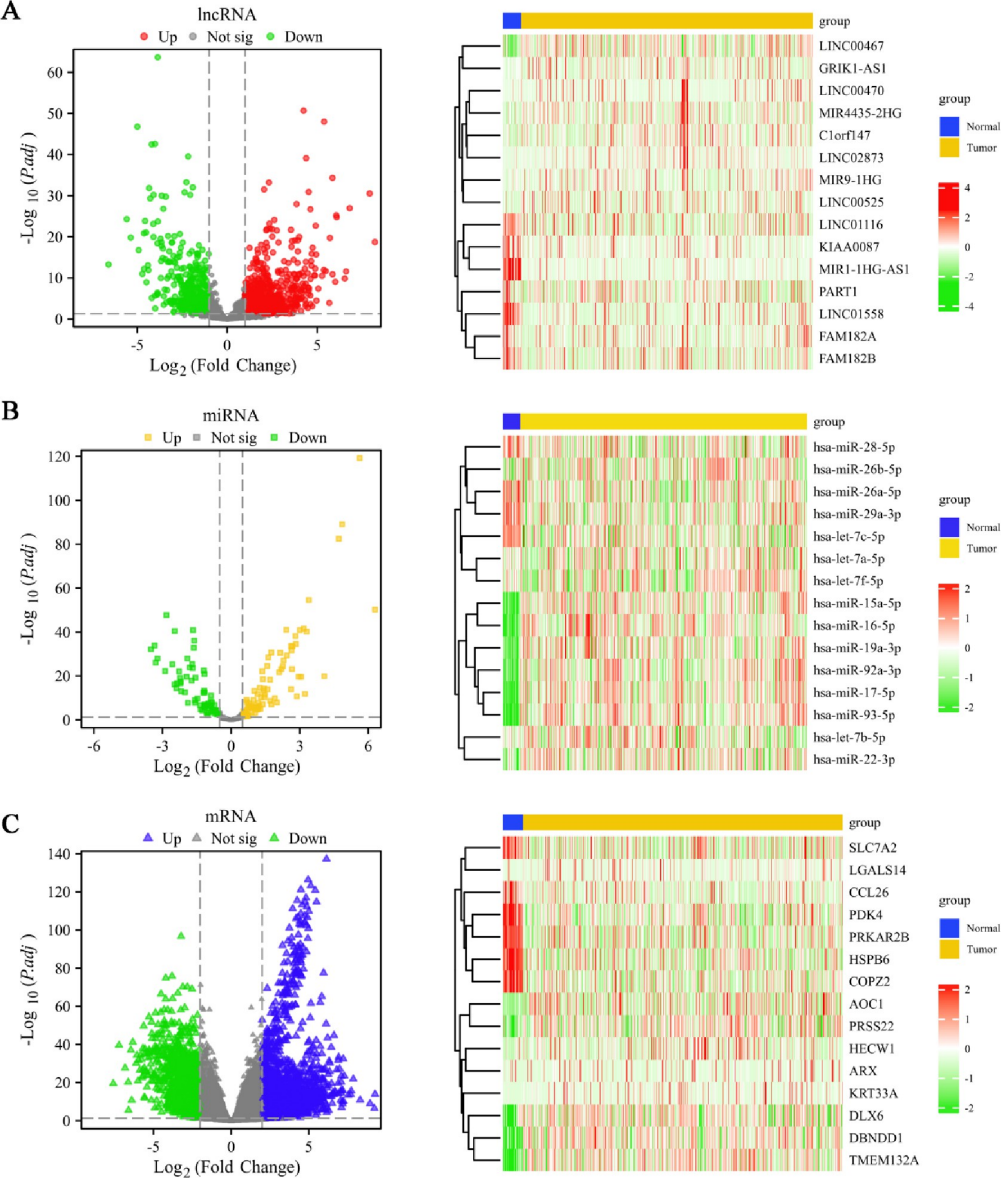
Differential expression analysis in UCEC. (**A**) Volcano plot of all DElncRNA genes in UCEC (|logFC| > 1 and p.adj < 0.05) and heatmap of the top 15 genes, (**B**) Volcano plot of all DEmiRNA genes in UCEC (|logFC| > 0.5 and p.adj < 0.05) and heatmap of the top 15 genes. (**C**) Volcano plot of all DEmRNA genes in UCEC (|logFC| > 2 and p.adj < 0.05) and heatmap of the top 15 genes.

In the analysis of 589 samples, we identified a significant number of differentially expressed genes. For lncRNA, a total of 1179 differentially expressed genes were identified, of which 758 were upregulated and 421 were downregulated (Fig 1A). For miRNAs, 151 differentially expressed genes were identified, with 85 upregulated and 66 downregulated (Fig 1B). For mRNAs, 2737 differentially expressed genes were identified, with 1722 upregulated and 1015 downregulated (Fig 1C). The top 15 differentially expressed genes for each category were visualized using heatmaps to highlight the most significant changes.

### Identification and Interaction Network of Prognostic lncRNAs, miRNAs, and mRNAs in UCEC

**Fig 2:**
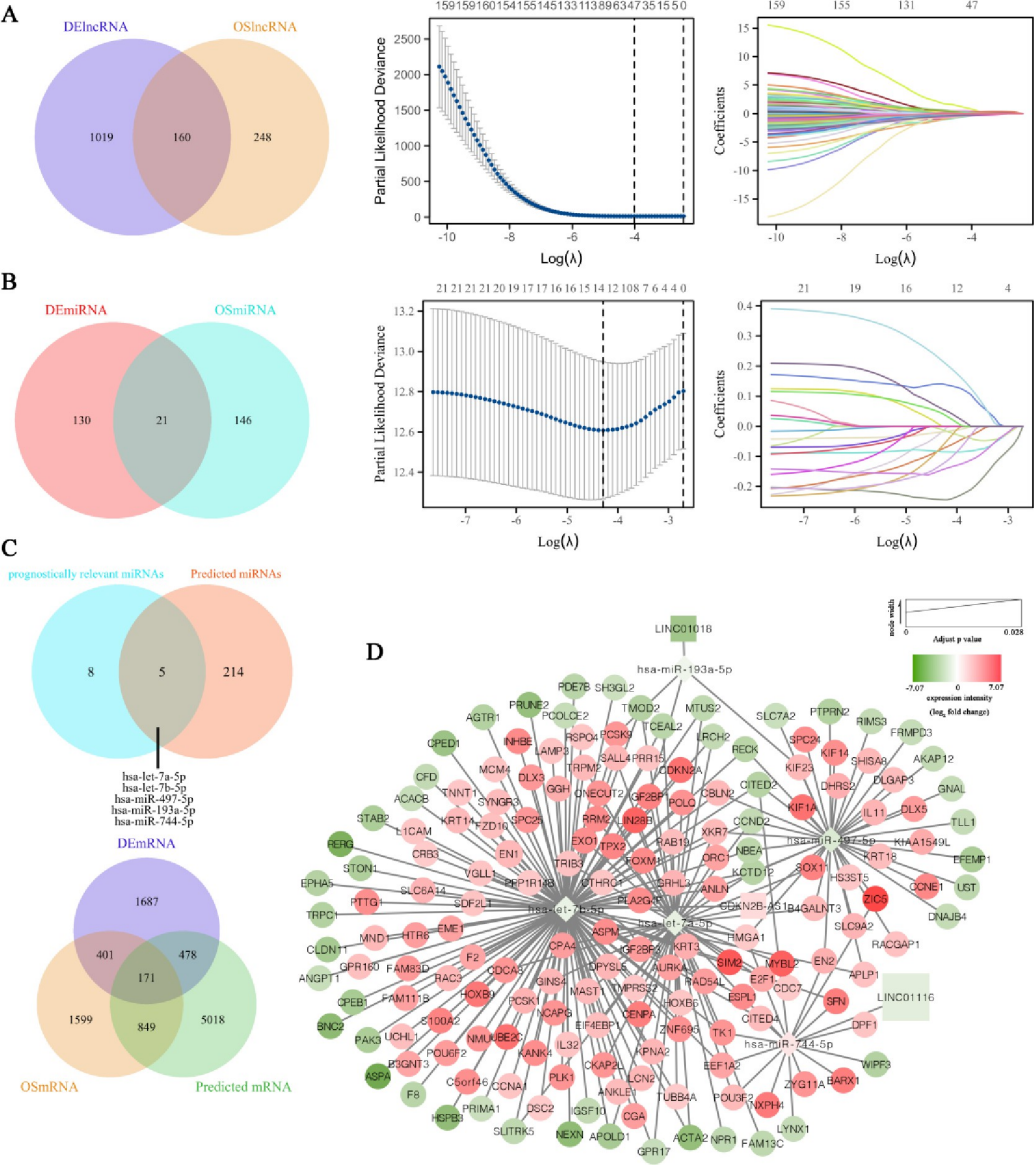
Prognostic lncRNAs, miRNAs, and mRNAs in UCEC. (**A**) Venn diagram showing 160 differentially expressed and OS-associated lncRNAs in UCEC. LASSO analysis selected 47 significant lncRNAs. (**B**) Venn diagram illustrating 21 overlapping differentially expressed and OS-associated miRNAs in UCEC. LASSO analysis selected 13 significant miRNAs. (**C**) Venn diagrams showing the overlap of miRNAs predicted by the 47 lncRNAs and the 13 miRNAs and the overlap of mRNAs predicted by these 5 miRNAs with differentially expressed and OS-associated mRNAs. (**D**) The triple regulatory network of lncRNA-miRNA-mRNA significantly associated with prognosis in UCEC. Nodes in red indicate upregulation, while nodes in green indicate downregulation. The size of each node is positively correlated with the adjusted p value.

We identified 160 overlapping lncRNAs that were both differentially expressed and associated with OS prognosis in UCEC. Using LASSO coefficient screening, we further narrowed these down to 47 significant lncRNAs, as demonstrated in the LASSO variable trajectory plot (Fig 2A). To similarly investigate miRNAs, we found 21 miRNAs that were differentially expressed and linked to OS prognosis in UCEC. LASSO coefficient analysis reduced this number to 13 critical miRNAs (Fig 2B). Through predictive analysis, we discovered 5 miRNAs (hsa-let-7a-5p, hsa-let-7b-5p, hsa-miR-497-5p, hsa-miR-193a-5p, and hsa-miR-744-5p) shared between the 47 identified lncRNAs and the 13 miRNAs. To further understand the relationship bewteen these miRNAs and mRNAs, we intersected the mRNAs predicted by these 5 miRNAs with DEmRNAs and OS-associated mRNAs, yielding 171 mRNAs (Fig 2C). Next, we constructed a comprehensive lncRNA-miRNAs-mRNA interaction network using Cytoscape, which includes 3 lncRNAs, 5miRNAs, and 171 mRNAs (Fig 2D). This network highlights the complex regulatory relationships and potential prognostic biomarkers in UCEC.

**Fig 3:**
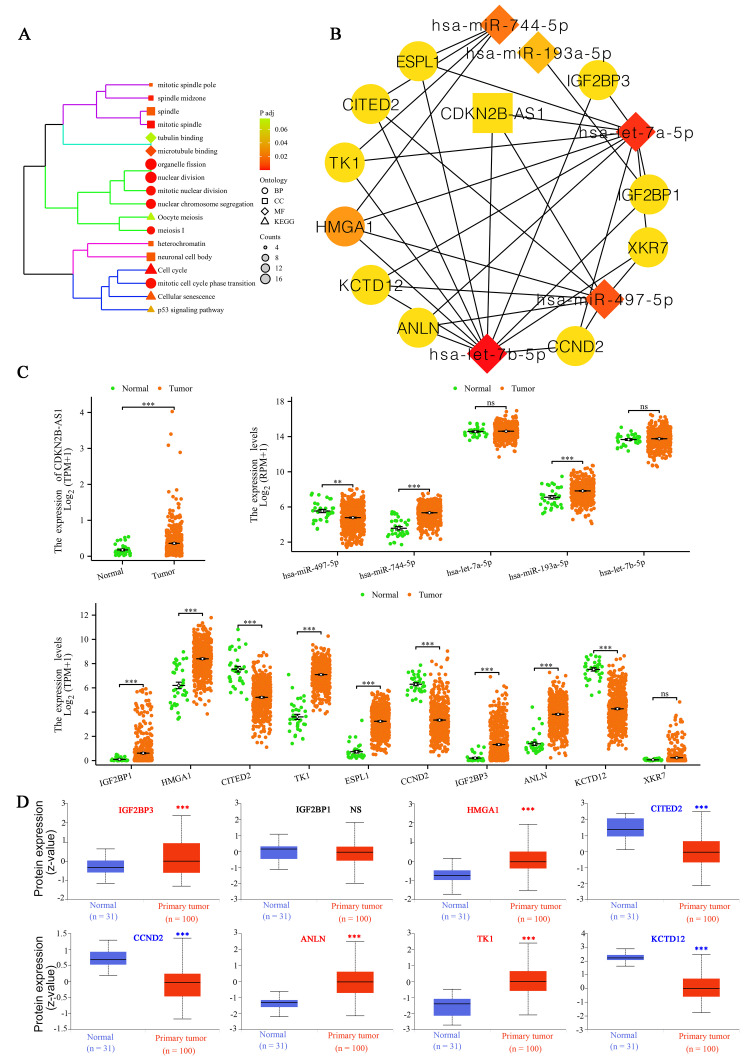
Analysis of the lncRNA-miRNA-mRNA network in UCEC. (**A**) GO and KEGG pathway analysis of mRNAs in the lncRNA-miRNA-mRNA network with a significance threshold of corrected p < 0.05. (**B**) Hub triple regulatory network identified using the Cytoscape plugin cytoHubba with a degree of > 3, including one lncRNA, five miRNAs, and ten mRNAs. (**C**) Differential expression of hub triple regulatory network RNAs in UCEC versus normal tissues. (**D**) Protein levels of the hub triple regulatory network in UCEC tissues. Notations: ns indicates no significance; **p < 0.01, ***p < 0.001.

To elucidate the biological significance of the identified mRNAs involved in the lncRNA-miRNA-mRNA network revealed significant enrichment in various biological processes (BP), cellular components (CC), and molecular functions (MF). Specifically, the BP category showed enrichment in nuclear division, organelle fission, nuclear chromosome segregation, mitotic cell cycle phase transition, mitotic nuclear division, and meiosis I. The CC category highlighted the mitotic spindle, spindle midzone, spindle, mitotic spindle pole, neuronal cell body, and heterochromatin. For the MF category, significant functions included microtubule binding and tubulin binding. KEGG pathway analysis indicated significant involvement in Cell Cycle, Cellular Senescence, p53 Signaling Pathway, and Oocyte Meiosis (Fig 3A). Using the Cytoscape plugin cytoHubba, we identified key components of the hub triple regulatory network, consisting of one lncRNA (CDKN2B-AS1), five miRNAs (hsa-miR-497-5p, hsa-miR-744-5p, hsa-let-7a-5p, hsa-miR-193a-5p, hsa-let-7b-5p), and ten mRNAs (IGF2BP1, HMGA1, CITED2, TK1, ESPL1, CCND2, IGF2BP3, ANLN, KCTD12, XKR7; Fig 3B). To validate the biological relevance of these components, we compared their expression patterns in UCEC tissues with those in normal endometrial tissues. UCEC tissues exhibited abnormal expression patterns in the identified RNAs of the hub triple regulatory network. Specifically, CDKN2B-AS1, hsa-miR-744-5p, hsa-miR-193a-5p, IGF2BP1, HMGA1, TK1, ESPL1, IGF2BP3, and ANLN were significantly upregulated, while hsa-miR-497-5p, CCND2, CITED2, and KCTD12 were significantly downregulated (Fig 3C). Further analysis at the protein levels in UCEC tissues revealed downregulation of CCND2, CITED2, and KCTD12, and upregulation of IGF2BP3, HMGA1, ANLN, and TK1 proteins. The expression of IGF2BP1 protein did not show significant differences when compared to normal tissues (Fig 3D).

**Fig 4:**
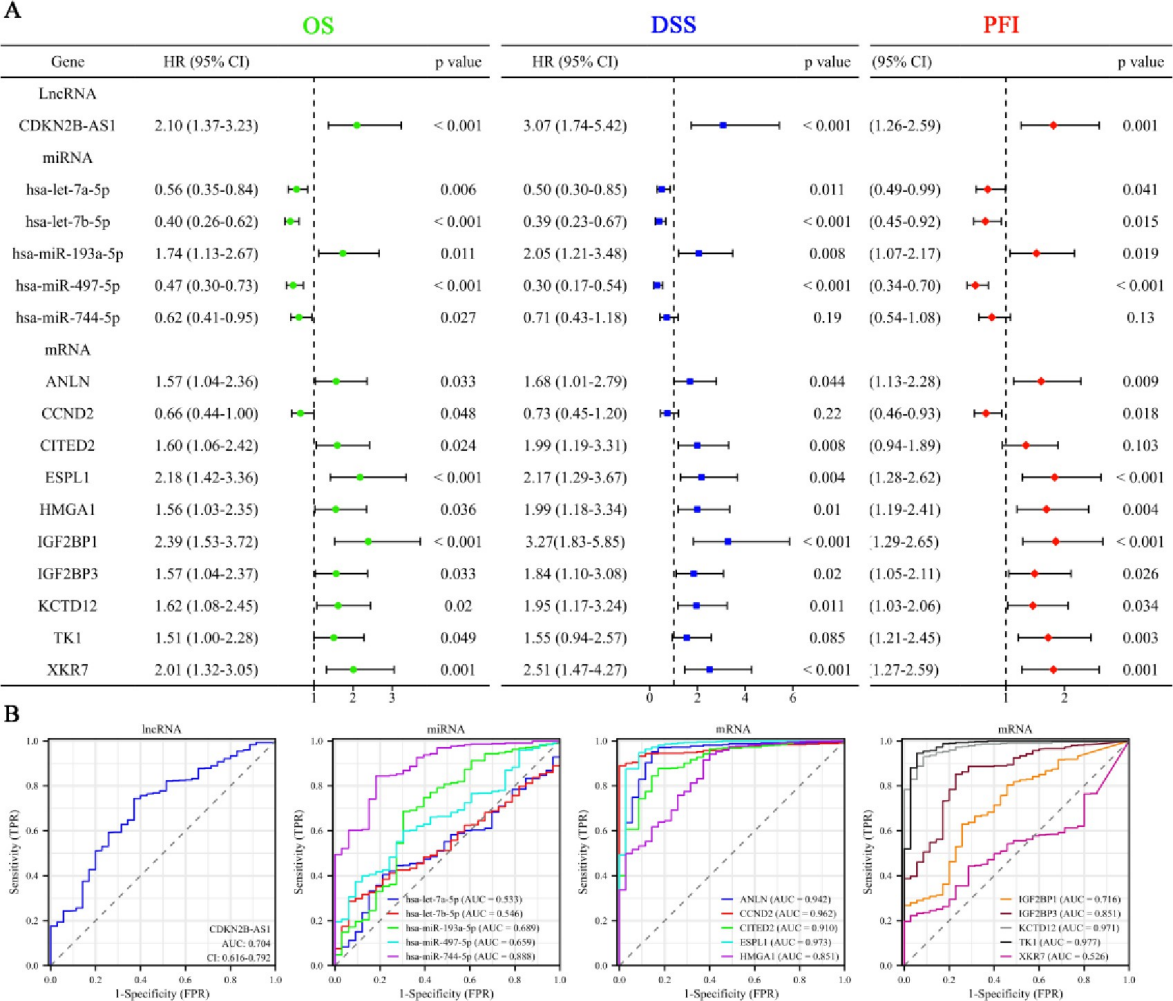
Survival and predictive analysis of hub triple regulatory network molecules in UCEC. (**A**) Forest plot showing the survival analysis (OS, DSS, and PFI) of hub triple regulatory network molecules in UCEC. (**B**) ROC analysis of hub triple regulatory network molecules.

We then performed survival analysis to assess the prognosis significance of these hub molecules. The forest plot illustrates the survival analysis outcomes (OS, DSS, and PFI) for the hub triple regulatory network molecules in UCEC. The analysis revealed that high expression levels of CDKN2B-AS1, hsa-miR-193a-5p, ANLN, ESPL1, HMGA1, IGF2BP3, IGF2BP1, KCTD12, and XKR7 are significantly associated with poor OS, DSS, and PFI prognosis. Conversely, high expression of hsa-let-7a-5p, hsa-let-7b-5p, and hsa-miR-497-5p correlates with a favorable prognosis (Fig 4A). To evaluate the predictive accuracy of these molecules, we performed ROC analysis calculated the AUC values for each hub molecule. The AUC values varied across different RNAs, with most showing moderate to high predictive accuracy. Specifically, CDKN2B-AS1 had an AUC of 0.704, indicating moderate predictive accuracy. Among the miRNAs, hsa-miR-497-5p and hsa-miR-193a-5p had AUC values of 0.659 and 0.689, respectively, indicating low accuracy. However, hsa-let-7a-5p and hsa-let-7b-5p showed relatively poor discriminatory ability, with AUC values of 0.533 and 0.546, respectively. For the mRNAs, several molecules exhibited high predictive accuracy. ESPL1 (AUC = 0.973), KCTD12 (AUC = 0.971), and ANLN (AUC = 0.942) all had AUC values above 0.9, indicating excellent predictive power. HMGA1 (AUC = 0.851) and IGF2BP3 (AUC = 0.851) also showed high accuracy. IGF2BP1 (AUC = 0.716) demonstrated moderate predictive accuracy, while XKR7 (AUC = 0.526) had relatively poor predictive performance (Fig 4B).

### Subcellular localization and regulatory interactions of CDKN2B-AS1 in UCEC

**Fig 5:**
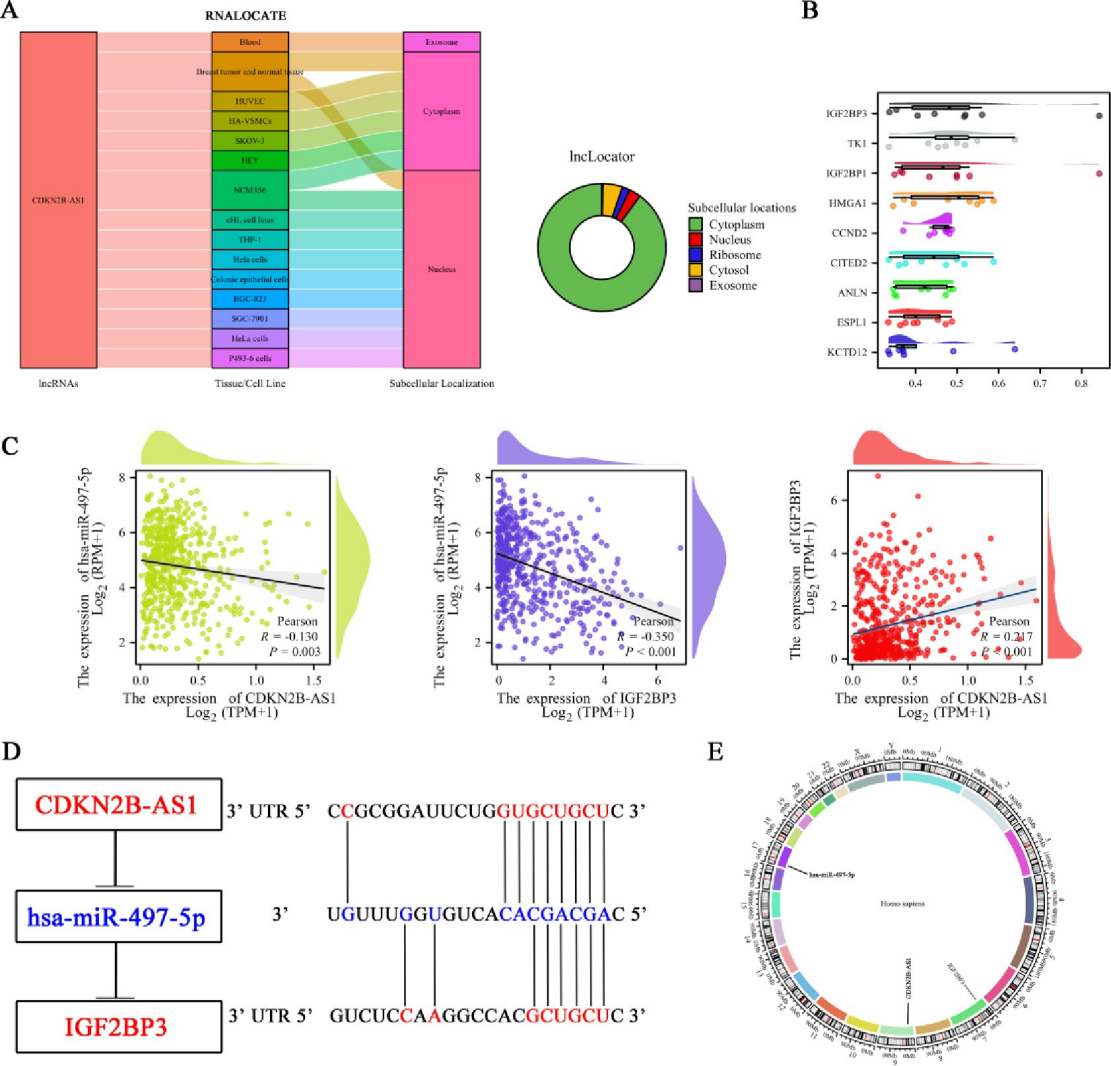
Subcellular localization and regulatory network of CDKN2B-AS1 in UCEC. (**A**) Subcellular localization of CDKN2B-AS1 primarily in the cytoplasm, as identified by lncLocator and A Sankey diagram illustrates the subcellular localization of CDKN2B-AS1 in various cells according to RNALOCATE. (**B**) Friends’ analysis identified IGF2BP3 as a key gene from the mRNAs in the hub triple regulatory network. (**C**) Correlation analysis involving CDKN2B-AS1, hsa-miR-497-5p and IGF2BP3 in UCEC. (**D**) Conceptual model of the ceRNA network CDKN2B-AS1-hsa-miR-497-5p-IGF2BP3, depicting predicted binding sites. Overexpressed genes are marked in red, and underexpressed genes are marked in blue. (**E**) Chromosomal location map displaying the positions of CDKN2B-AS1, hsa-miR-497-5p, and IGF2BP3 on their respective chromosomes.

To determine the subcellular localization of CDKN2B-AS1, analysis using lncLocator revealed that CDKN2B-AS1 is predominantly localized in the cytoplasm. This finding was further confirmed by RNALOCATE, which showed that CDKN2B-AS1 is distributed in the cytoplasm of various cell types including breast tumor and normal tissue, HUVEC, HA-VSMCs, SKOV-3, HEY, and NCM356 (Fig 5A). Identifying the key genes in the hub triple regulatory network is crucial for understanding their potential regulatory roles. Using Friends analysis, IGF2BP3 was identified as a key gene among the mRNAs in the hub triple regulatory network, highlighting its potential regulatory importance (Fig 5B). To explore the regulatory relationships further, expression correlation analysis was performed, indicated that both CDKN2B-AS1 and IGF2BP3 are negatively correlated with hsa-miR-497-5p. Additionally, a positive correlation was observed between the expression of CDKN2B-AS1 and IGF2BP3, suggesting a possible regulatory interaction within this network (Fig 5C). Based on these findings, a conceptual ceRNA network model CDKN2B-AS1-hsa-miR-497-5p-IGF2BP3 was constructed illustrating the predicted binding sites (Fig 5D). To provide a comprehensive understanding of their genomic context and potential interactions, the chromosomal location map provides the precise positions of CDKN2B-AS1, hsa-miR-497-5p, and IGF2BP3 on their respective chromosomes, aiding in the understanding of their genomic context and potential interactions (Fig 5E). To further validate these findings, GEO analysis showed hsa-miR-744-5p was significantly downregulated in UCEC in the GSE35794 dataset, consistent with our results. In the GSE106191 dataset, IGF2BP3 was significantly upregulated in UCEC, while CDKN2B-AS1 showed no differential expression (S1 Fig).

### Prognostic analysis of CDKN2B-AS1, IGF2BP3, and hsa-miR-497-5p in UCEC

**Fig 6:**
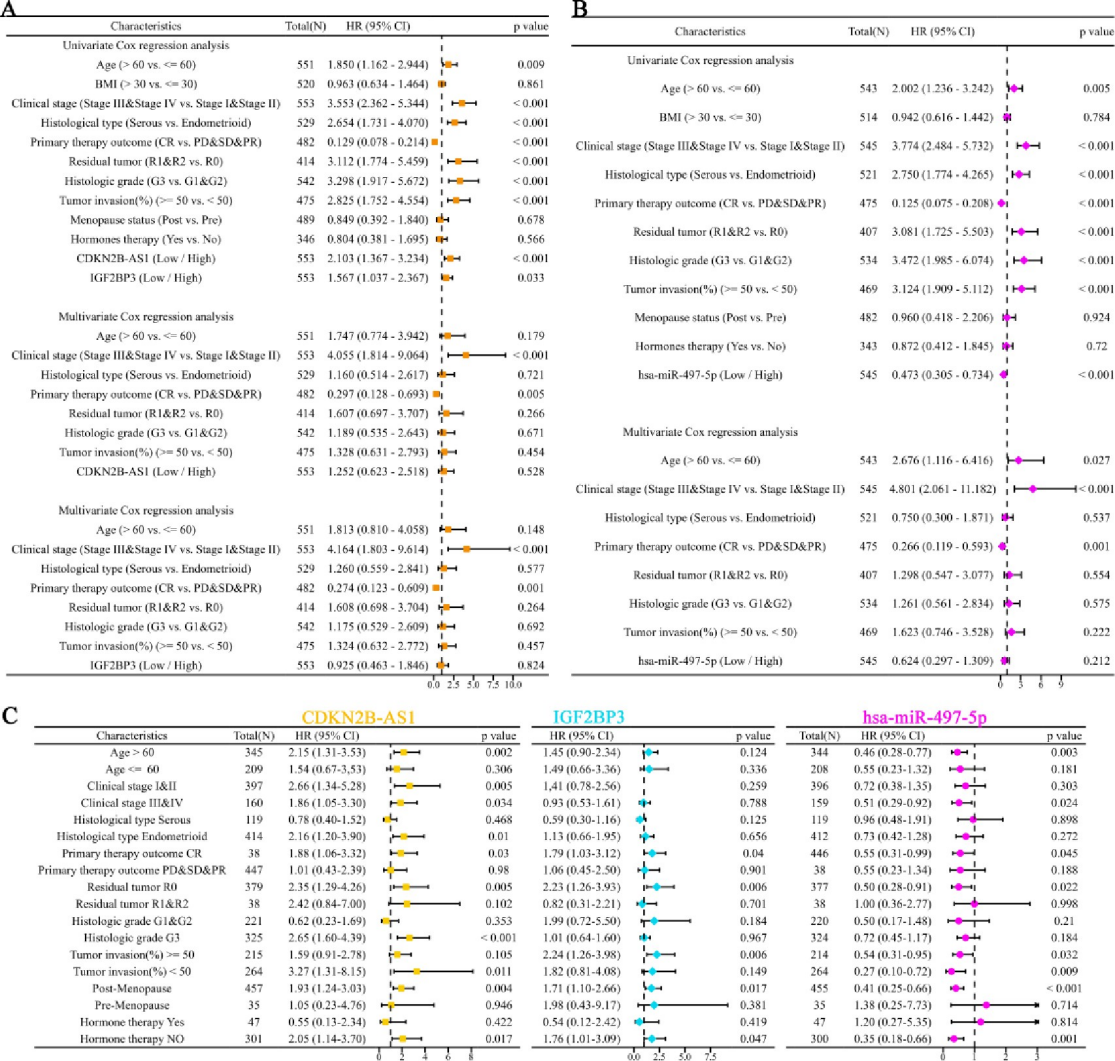
Prognostic analysis of CDKN2B-AS1, IGF2BP3, and hsa-miR-497-5p in UCEC. (**A**) Univariate and multivariate Cox regression analyses of CDKN2B-AS1 and IGF2BP3. (**B**) Univariate and multivariate Cox regression analyses of hsa-miR-497-5p. (**C**) Comparison of prognosis among different subgroups based on the expression levels of CDKN2B-AS1, IGF2BP3, and hsa-miR-497-5p.

To assess the prognostic significance of CDKN2B-AS1 and IGF2BP3, univariate and multivariate Cox regression analyses were performed. In the univariate Cox regression model, several prognostic factors, including Age, Clinical stage, Histological type, Primary therapy outcome, Residual tumor, Histologic grade, and Tumor invasion, were significantly associated with OS in UCEC patients (p < 0.05). Notably, overexpression of CDKN2B-AS1 (HR = 2.103, p < 0.001) and IGF2BP3 (HR = 1.567, p = 0.033) was significantly associated with poor prognosis. Conversely, low expression of hsa-miR-497-5p (HR = 0.473, p < 0.001) was associated with poor prognosis. However, to determine the independent prognostic value of these factors, multivariate Cox regression analysis was conducted, revealing that only Clinical stage and Primary therapy outcome remained significantly associated with OS, while the expression levels of CDKN2B-AS1, IGF2BP3, and hsa-miR-497-5p were not significantly associated with OS (Fig 6A and 6B). To provide more detailed prognostic information, we compared prognosis among different subgroups based on the expression levels of CDKN2B-AS1, IGF2BP3, and hsa-miR-497-5p. We observed that CDKN2B-AS1, IGF2BP3, and hsa-miR-497-5p exhibited good prognostic abilities in subgroups such as Primary therapy outcome CR, Residual tumor R0, Post-Menopause, and Hormone therapy NO (Fig 6C). To visualize and estimate individual patients’ prognosis, we developed a nomogram to predict the 1-, 3-, and 5-year survival rates of UCEC patients based on several factors, including Age, BMI, Clinical stage, Histological type, Primary therapy outcome, Residual tumor, Histologic grade, Tumor invasion, Menopause status, Hormone therapy, CDKN2B-AS1, IGF2BP, and hsa-miR-497-5p. This nomogram provides a visual to estimate individual patient prognosis by assigning a score to each factor and calculating the total score to predict survival probabilities (S2 Fig).

### Differential expression and methylation analysis of IGF2BP3 in UCEC

**Fig 7:**
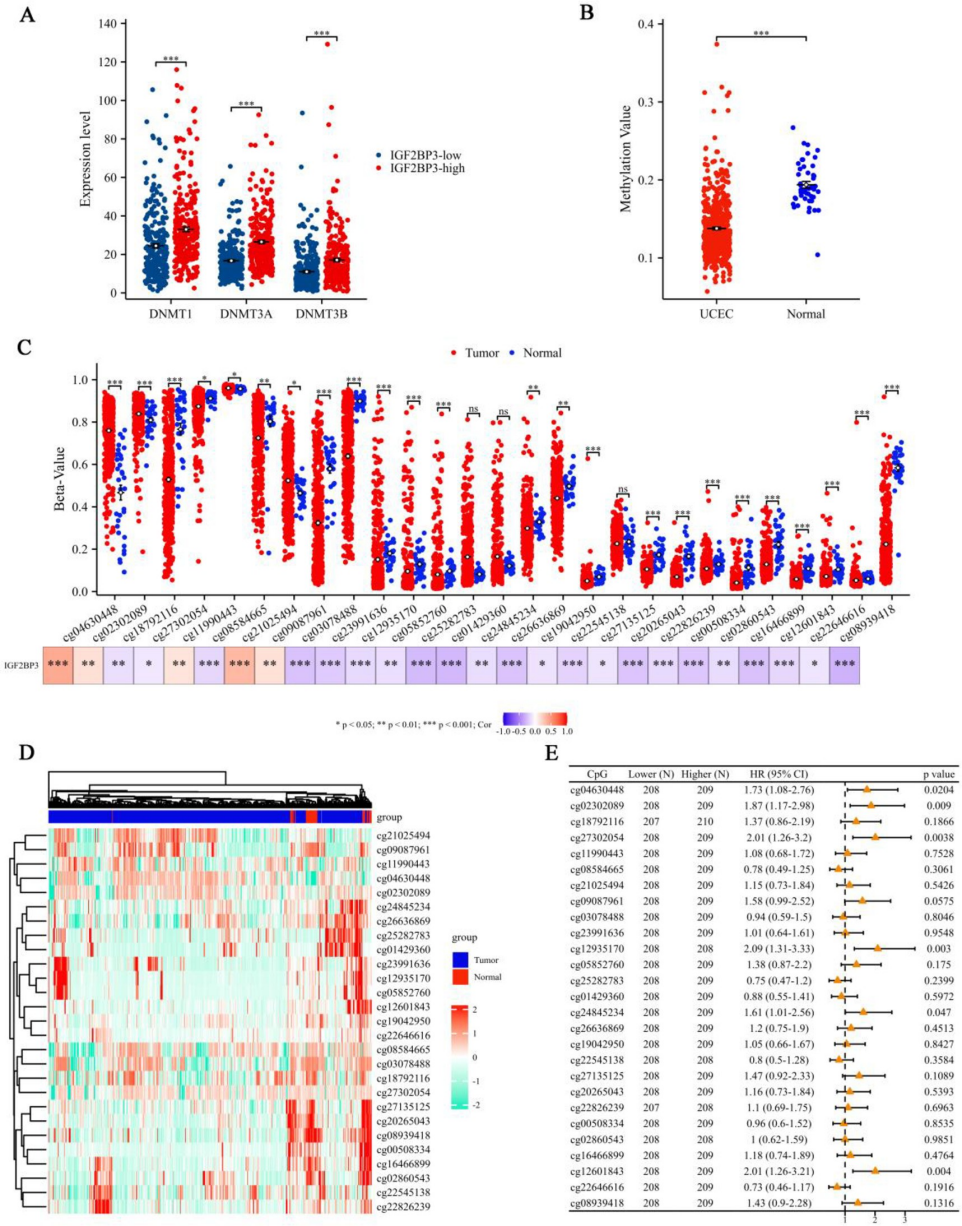
Methylation analysis of IGF2BP3 in UCEC. (**A**) Comparison of the expression levels of three DNA methyltransferases (DNMT1, DNMT3A, and DNMT3B) between UCEC samples with high and low IGF2BP3 expression. (**B**) Analysis of IGF2BP3 methylation status in UCEC using the DiseaseMeth 2.0 tool. (**C**) Differences in the methylation intensity of IGF2BP3 CpGs between UCEC and normal tissue samples, and the correlation between methylation values at various CpG sites and IGF2BP3 expression in UCEC. (**D**) Heatmap representing the clustering of CpG methylation levels with IGF2BP3 in UCEC. (**E**) Forest plot illustrating the impact of different CpG site methylation levels within IGF2BP3 on survival in UCEC. Notations: ns indicates no significance; *p < 0.05, **p < 0.01, ***p < 0.001.

To explore the molecular mechanisms underlying IGF2BP3 expression, we compared the expression levels of DNMT1, DNMT3A, and DNMT3B between UCEC samples with high and low IGF2BP3 expression. All these DNA methyltransferases showed significantly higher expression levels in UCEC tissues with high IGF2BP3 expression (Fig 7A). Given the importance of DNA methylation in gene regulation, we analyzed the methylation status of IGF2BP3 in UCEC using the DiseaseMeth 2.0 tool, revealing that IGF2BP3 exhibited low methylation levels in UCEC (Fig 7B). To further understand the relationship between methylation and gene expression, we investigated the differences in methylation intensity of CpGs within IGF2BP3 between UCEC and normal tissue samples and studied the correlation between methylation values at various CpG sites and IGF2BP3 expression in UCEC. The beta-values of cg04630448, cg02302089, cg11990443, and cg21025494 were significantly upregulated and positively correlated with IGF2BP3 expression. Conversely, the beta-values of cg18792116, cg27302054, cg08584665, cg03078488, cg12935170, cg05852760, cg24845234, cg26636869, cg19042950, cg27135125, cg20265043, cg22826239, cg00508334, cg02860543, cg16466899, cg12601843, cg22646616, and cg08939418 were significantly downregulated and negatively correlated with IGF2BP3 expression (Fig 7C). To visualize these findings, a heatmap representing the clustering of CpG methylation levels within IGF2BP3 in UCEC was generated (Fig 7D). To assess the clinical relevance of these methylation changes, we illustrated the impact of different CpG site methylation levels within IGF2BP3 on survival in UCEC was illustrated using a forest plot. High methylation levels of cg04630448, cg02302089, cg27302054, cg12935170, cg24845234, and cg12601843 were associated with lower OS rates in UCEC (Fig 7E).

### Immune infiltration and potential therapeutic analysis in UCEC

**Fig 8:**
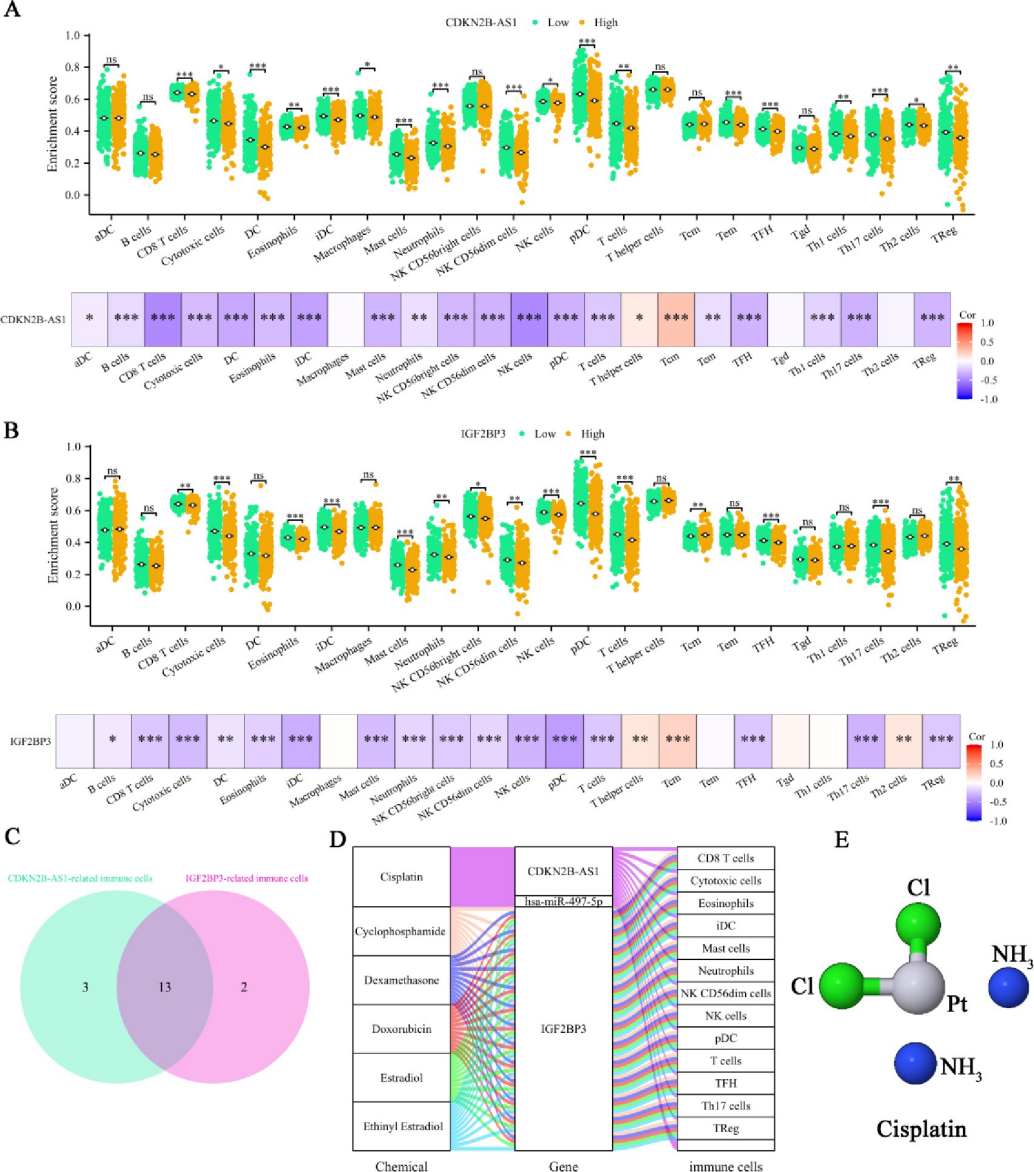
Immune infiltration and potential therapeutic analysis. Differential enrichment scores of 24 immune cell types and the correlation between gene expression and the proportion of 22 immune cell types in UCEC between the high and low CDKN2B-AS1 (**A**) and IGF2BP3 (**B**)expression groups. (C) Venn diagram showing immune cells affected by the expression of both CDKN2B-AS1 and IGF2BP3. (**D**) Sankey diagram illustrating the flow between candidate drugs, genes, and immune cells. (**E**) 3D structure of Cisplatin, a candidate drug for treating UCEC through its impact on the ceRNA network. Notations: ns indicates no significance; *p < 0.05, **p < 0.01, ***p < 0.001.

To understanding the immune landscape of UCEC, we compared to normal tissues, UCEC tissues exhibited higher enrichment scores for aDCs, NK CD56dim cells, TFH cells, Th2 cells, and regulatory T cells (Tregs) cells, while showing lower enrichment cores for CD8 T cells, cytotoxic cells, DCs, eosinophils, mast cells, neutrophils, NK cells, plasmacytoid DCs (pDCs), T helper cells, central memory T cells (Tcm), effector memory T cells (Tem), gamma delta T cells (Tgd), and Th1 cells (S3 Fig). Additionally, UCEC samples displayed higher immuneScores but lower StromaScores and MicroenvironmentScores compared to normal tissues (S3 Fig). To delve deeper into the immune microenvironment, correlation analysis of 22 immune cell types in UCEC revealed significant associations, such as a negative correlation between T cells CD8 and T cells CD4 memory resting, Macrophages M0, and Dendritic cells activated (S3 Fig). Using the CIBERSORT core algorithm, samples were stratified into high and low expression groups based on median expression levels of CDKN2B-AS1 and IGF2BP3. A stacked bar plot was utilized to visually compare immune cell scores across samples (S3 Fig). To further investigate the immune landscape, we compared the differential enrichment score of 24 immune cell types and analyzed the correlation between CDKN2B-AS1 expression and the proportion of 22 immune cell types in UCEC. In the high CDKN2B-AS1 expression group, we observed significantly lower enrichment scores for CD8 T cells, cytotoxic cells, DCs, eosinophils, immature DCs (iDCs), macrophages, mast cells, neutrophils, NK CD56dim cells, NK cells, plasmacytoid DCs (pDCs), T cells, Tem, follicular helper T cells (TFH), Th1 cells, Th17 cells, Th2 cells, and Tregs. Except for macrophages and Th2 cells, these immune cell proportions were negatively correlated with CDKN2B-AS1 expression in UCEC (Fig 8A). Similarly, the high IGF2BP3 expression group exhibited lower enrichment scores for CD8 T cells, cytotoxic cells, eosinophils, iDCs, mast cells, neutrophils, NK CD56bright cells, NK CD56dim cells, NK cells, pDCs, T cells, TFH, Th17 cells, and Tregs, with a negative correlation to IGF2BP3 expression. Notably, Tcm had higher enrichment scores in the high IGF2BP3 expression group and were positively correlated with IGF2BP3 expression (Fig 8B). To identify the common immune cells influenced by both genes, Intersection analysis revealed that 13 immune cell types were affected by the expression of both CDKN2B-AS1 and IGF2BP3 expression (Fig 8C). Small molecule drugs with potential therapeutic effects on UCEC were identified through the ceRNA network, and their interactions with genes and immune cells were visualized. IGF2BP3 had relatively abundant targeted drugs compared to CDKN2B-AS1 and hsa-miR-497-5p. Cisplatin was identified as a drug that targets both CDKN2B-AS1 and hsa-miR-497-5p and interacts with multiple immune cells, suggesting these immune cells as potential therapeutic targets for UCEC (Fig 8D). To provide a structural basis for its potential efficacy, the 3D structure of Cisplatin, a candidate drug for treating UCEC through its impact on the ceRNA network, was displayed (Fig 8E).

### Genetic and functional analysis of IGF2BP3 in UCEC

**Fig 9:**
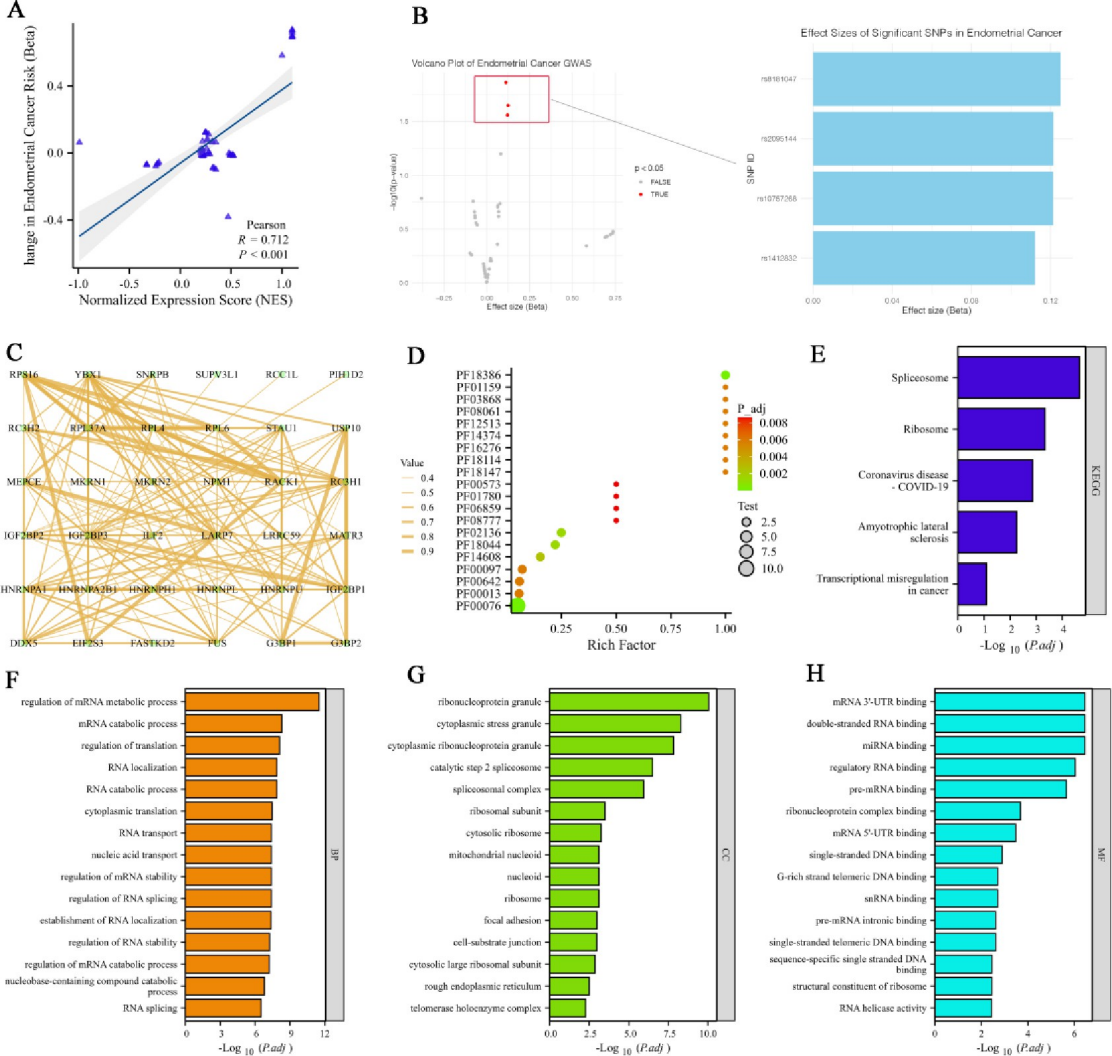
Genetic and functional analysis of IGF2BP3 in UCEC. (**A**) Correlation analysis of SNPs affecting IGF2BP3 expression with the risk of endometrial cancer. (**B**) Effect size of significant SNPs in endometrial cancer. (**C**) Correlation analysis of 35 experimentally validated IGF2BP3-binding proteins in UCEC. (**D**) Domain enrichment analysis of IGF2BP3-binding proteins. (**E**) KEGG pathway analysis based on IGF2BP3-associated genes. GO enrichment analysis for IGF2BP3-associated genes distributed across Cellular Component (**F**), Biological Process (**G**), and Molecular Function (**H**).

To further explore the molecular mechanisms underlying IGF2BP3’s role in UCEC, we analyzed the association between SNPs affecting IGF2BP3 expression and the risk of endometrial cancer (Fig 9A). This analysis identified 4 SNPs significantly associated with UCEC, including rs10757268, rs1412832, rs2095144, and rs8181047 (Fig 9B), suggesting these SNPs might modulate IGF2BP3 expression, thereby contributing to cancer risk. Next, to understand the protein-level interactions of IGF2BP3, we conducted a correlation analysis of 35 experimentally validated IGF2BP3-binding proteins in UCEC samples. The analysis revealed the protein-protein interaction network, highlighting the interconnected nature of these proteins in the context of UCEC (Fig 9C). This network provides insights into the potential regulatory roles of IGF2BP3-binding proteins. To further characterize the functional significance of these proteins, we performed a domain enrichment analysis of IGF2BP3-binding proteins. The analysis showed a significant enrichment of the PF00076 domain, indicating that this domain is crucial in the functional landscape of these proteins and suggesting a specific role for IGF2BP3-related proteins in UCEC (Fig 9D). Additionally, KEGG pathway analysis was conducted on IGF2BP3-associated genes to identify the biological pathways involved. The analysis demonstrated that IGF2BP3 is involved in transcriptional dysregulation pathways, which are crucial in the occurrence and development of UCEC (Fig 9E). To gain a more comprehensive understanding of the functional roles of IGF2BP3-associated genes, we performed GO enrichment analyses across BP, CC, and MF. These results indicated that IGF2BP3-related genes are predominantly enriched in the regulation of mRNA metabolic processes, RNA processing and transport, as well as immune regulation and cell differentiation (Fig 9F–9H).

## Discussion

UCEC is one of the most common estrogen-dependent cancers in women, with poor survival rates for advanced cases [18,19]. Non-coding RNAs have been implicated in UCEC progression, but their precise molecular mechanisms require further investigation. In this study, we constructed a ceRNA network linked to UCEC prognosis, identifying 1,179 lncRNAs, 151 miRNAs, and 2,737 mRNAs. Through LASSO regression and survival analysis, we pinpointed a core network of 3 lncRNAs, 5 miRNAs, and 171 mRNAs, primarily involved in cell cycle regulation and genomic stability. We identified a regulatory module centered around CDKN2B-AS1, 5 miRNAs, and 10 mRNAs. Subcellular localization analysis confirmed CDKN2B-AS1’s cytoplasmic distribution, supporting its role in ceRNA regulation. IGF2BP3 was identified as the key mRNA regulated by CDKN2B-AS1, with a strong regulatory relationship involving hsa-miR-497-5p. While multivariate analysis indicated that clinical stage and therapy outcome were significant prognostic factors, the CDKN2B-AS1-hsa-miR-497-5p-IGF2BP3 axis remained an important prognostic indicator, particularly in specific subgroups. This highlights the potential of this axis as a complementary biomarker for personalized prognosis and treatment in UCEC.

CDKN2B-AS1is a recently discovered lncRNA located in the CDKN2B-CDKN2A gene cluster on chromosome 9p21. It is significantly upregulated in various cancers and functions as a ceRNA, sponging miRNAs to regulate target mRNAs, thereby promoting processes such as cell proliferation, apoptosis, and migration [20]. Consistent with studies in other cancers, such as thyroid cancer [21] and ovarian cancer [22]. our results demonstrate that CDKN2B-AS1 is highly expressed in UCEC and is associated with poor prognosis, underscoring its likely involvement in UCEC progression. However, in our validation using the GSE106191 dataset, CDKN2B-AS1 did not show significant differential expression in UCEC. This discrepancy could stem from differences in sample size, cohort characteristics, or technical factors such as probe design, as GSE106191 may not be optimized for non-coding RNA detection. Despite this inconsistency, our findings are supported by previous research [23], reaffirming the role of CDKN2B-AS1 in UCEC. Furthermore, we found that high CDKN2B-AS1 expression correlates with lower immune cell infiltration in UCEC, suggesting that it may contribute to immune evasion and worse clinical outcomes. Thus, CDKN2B-AS1 not only serves as a potential prognostic biomarker but also represents a novel therapeutic target in UCEC.

While hsa-miR-497-5p is downregulated in several cancers, including non-small cell lung carcinoma Cells [24], liver cancer [25], gastric cancer [26], and endometrial cancer [27], our study also identified hsa-miR-497-5p as significantly underexpressed in UCEC, where its low expression is associated with poor prognosis. This is consistent with prior studies demonstrating its tumor-suppressive role in UCEC through the hsa-miR-497-5p/FASN axis [28]. The low expression of hsa-miR-497-5p in our ceRNA network further highlights its importance in regulating key oncogenic pathways in UCEC.

Insulin-like growth factor 2 mRNA-binding protein 3 (IGF2BP3) has been shown to promote cancer progression across various tumor types [29–34]. In UCEC, our findings align with previous research [35], demonstrating that IGF2BP3 is significantly overexpressed and associated with poor survival outcomes. Furthermore, our study is the first to show that high IGF2BP3 expression correlates with elevated DNA methyltransferases (DNMT1, DNMT3A, DNMT3B), suggesting a potential link between epigenetic regulation and IGF2BP3 expression in UCEC. The lower methylation levels of IGF2BP3 CpG sites may drive its overexpression, similar to mechanisms observed in gliomas [36].

Our immune cell analysis revealed significantly lower infiltration of cytotoxic immune cells, such as CD8+ T cells, in UCEC tissues with high IGF2BP3 expression. This supports the hypothesis that IGF2BP3 may contribute to immune evasion by modulating immune checkpoints, such as PD-L1 [37]. Furthermore, our drug interaction analysis suggests that IGF2BP3 may be targeted by various small molecule drugs, opening up possibilities for therapeutic interventions. These findings highlight the importance of the immune landscape in UCEC treatment strategies, particularly in the context of IGF2BP3 expression.

In addition to expression analyses, our SNP analysis identified significant correlations between IGF2BP3 SNPs (e.g., rs10757268 and rs1412832) and increased risk for UCEC, suggesting a genetic predisposition linked to IGF2BP3 dysregulation. Moreover, our protein interaction network analysis uncovered interactions between IGF2BP3 and several key proteins involved in mRNA metabolism, immune regulation, and cell differentiation. These findings, consistent with other studies in liver cancer [32], underscore the complex regulatory role of IGF2BP3 in UCEC and its potential as a therapeutic target.

In this study, we introduce key innovations in ceRNA network research for UCEC. Using a large-scale TCGA dataset, we provide a more comprehensive and statistically robust assessment of ceRNA interactions. By integrating immune cell infiltration, DNA methylation, and SNP analyses, we offer novel insights into the epigenetic and immunological regulation of UCEC, identifying potential biomarkers and therapeutic targets. Despite our pioneering construction of the CDKN2B-AS1-hsa-miR-497-5p-IGF2BP3 axis as a potential prognostic biomarker for clinical application, certain limitations must be acknowledged. Firstly, the binding affinities of the lncRNAs, miRNAs, and mRNAs retrieved from databases require experimental validation. Secondly, the functions and mechanisms of the CDKN2B-AS1/IGF2BP3 axis in UCEC initiation and progression need further exploration through in vitro and in vivo studies. Additionally, using TCGA data poses potential limitations such as lack of global diversity, incomplete clinical data, bath effects, and inability to fully capture tumor heterogeneity, despite our standardization efforts.

## Conclusion

In conclusion, our study identified the CDKN2B-AS1-hsa-miR-497-5p-IGF2BP3 ceRNA axis as a significant prognostic factor in UCEC. This network is linked to poor prognosis and influences immune cell infiltration and DNA methylation in UCEC tissues. The high expression of CDKN2B-AS1 and IGF2BP3, along with the low expression of hsa-miR-497-5p, suggests their potential as prognostic biomarkers and therapeutic targets. Our findings emphasize the role of the tumor environment and provide a basis for future research into immune-based therapies. Overall, the CDKN2B-AS1-hsa-miR-497-5p-IGF2BP3 axis offers new insights into UCEC molecular mechanisms and could lead to improved diagnostic and treatment strategies for UCEC. Further validation and mechanistic studies are necessary to fully explore its therapeutic potential.

## Supporting information

S1 Fig Validated of ceRNA network in GSE106191 and GSE35794. (TIF)

S2 Fig Diagnostic nomogram of ceRNA. (TIF)

S3 Fig Immune landscape of UCEC. (TIF)
