# Supplementary figures and images for: Comprehensive analysis of ceRNA Networks in UCEC: Prognostic and therapeutic implications
Source: PLoS One. 2025 Jan 30;20(1):e0314314. doi: 10.1371/journal.pone.0314314 (PMC11781699; doi:10.1371/journal.pone.0314314)

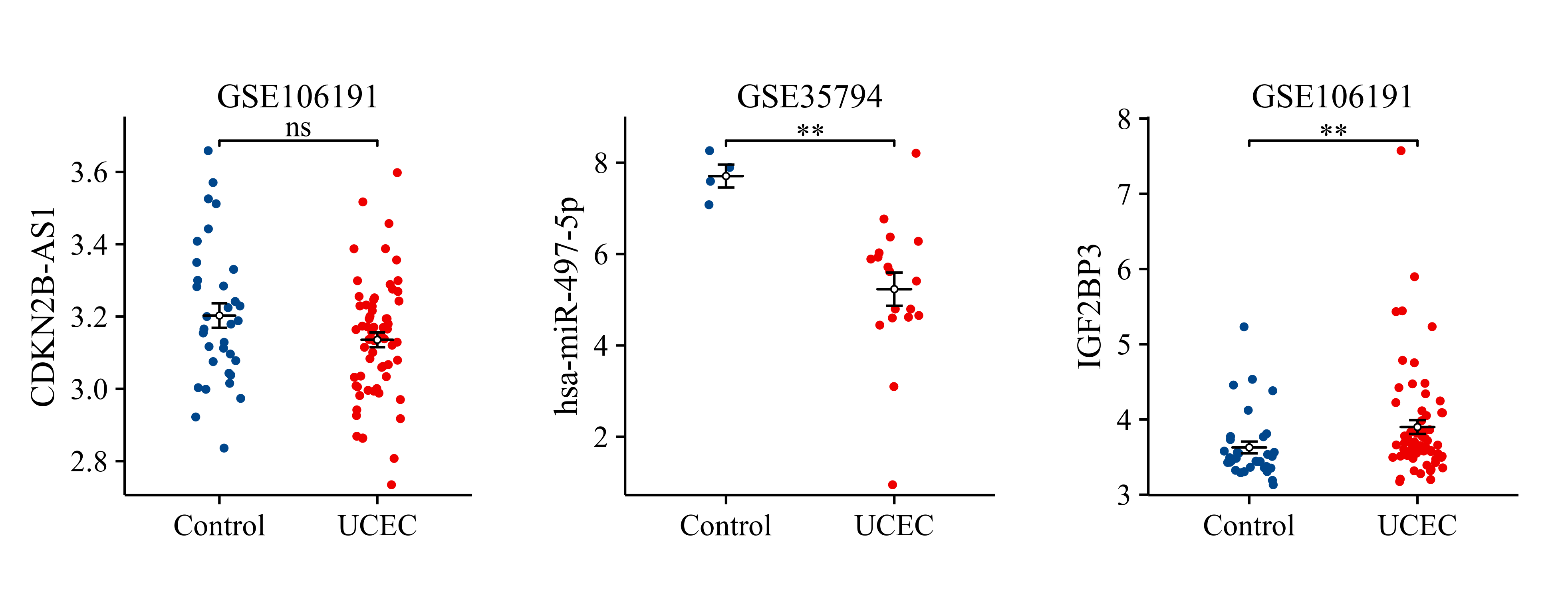

Supplement: S1 Fig — (TIF) [file pone.0314314.s001.tif]

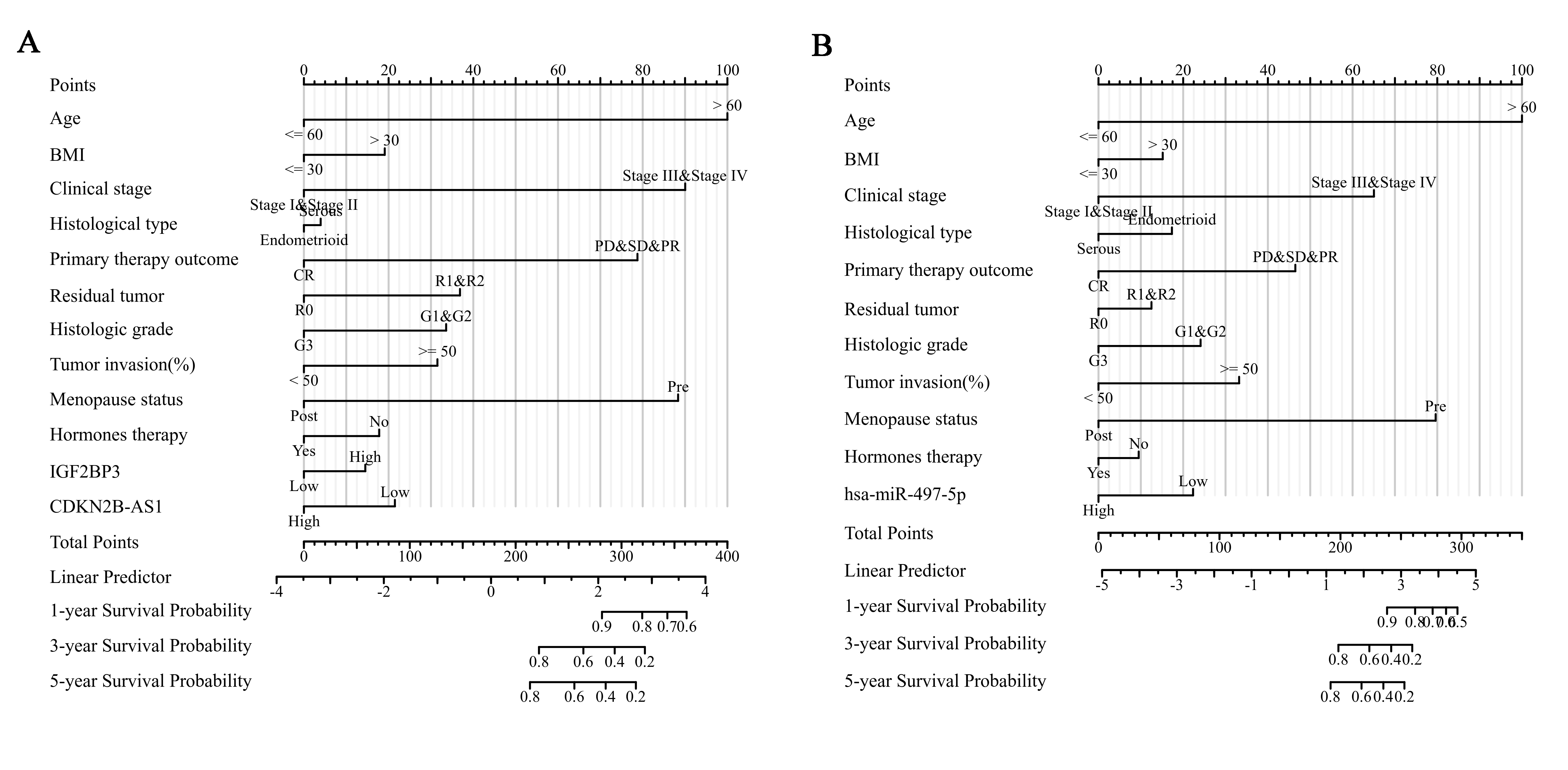

Supplement: S2 Fig — (TIF) [file pone.0314314.s002.tif]

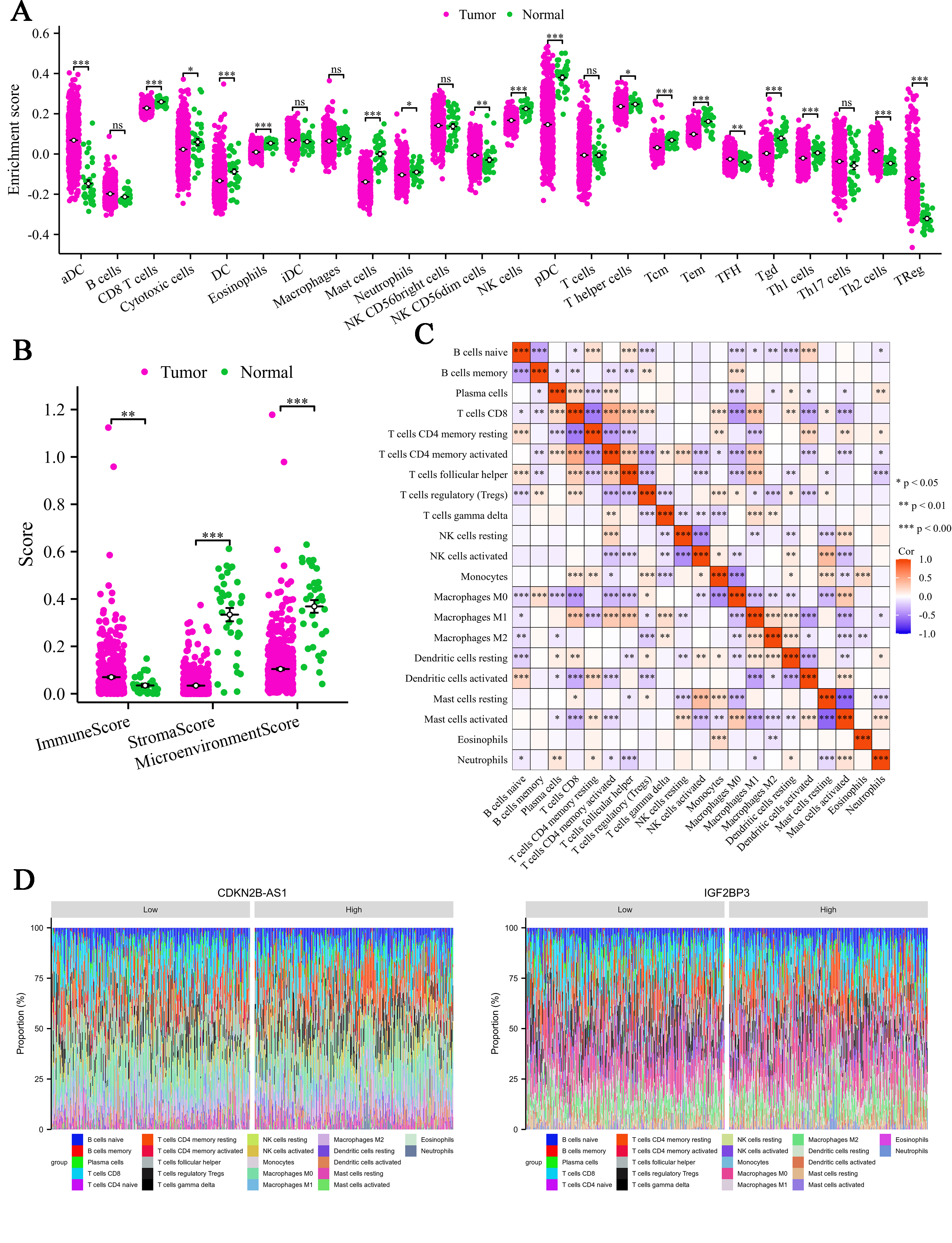

Supplement: S3 Fig — (TIF) [file pone.0314314.s003.tif]
